# Structural Changes of Amyloid Beta in Hippocampus of Rats Exposed to Ozone: A Raman Spectroscopy Study

**DOI:** 10.3389/fnmol.2017.00137

**Published:** 2017-05-22

**Authors:** Selva Rivas-Arancibia, Erika Rodríguez-Martínez, Isidro Badillo-Ramírez, Ulises López-González, José M. Saniger

**Affiliations:** ^1^Departamento de Fisiología, Facultad de Medicina, Universidad Nacional Autónoma de MéxicoCiudad de México, Mexico; ^2^Centro de Ciencias Aplicadas y Desarrollo Tecnológico, Universidad Nacional Autónoma de MéxicoCiudad de México, Mexico

**Keywords:** oxidative stress, Raman spectroscopy, amyloid beta 1–42, ozone, neurodegeneration, Alzheimer’s disease

## Abstract

The aim of this work was to study the effect of oxidative stress on the structural changes of the secondary peptide structure of amyloid beta 1–42 (Aβ 1–42), in the dentate gyrus of hippocampus of rats exposed to low doses of ozone. The animals were exposed to ozone-free air (control group) and 0.25 ppm ozone during 7, 15, 30, 60, and 90 days, respectively. The samples were studied by: (1) Raman spectroscopy to detect the global conformational changes in peptides with α-helix and β-sheet secondary structure, following the deconvolution profile of the amide I band; and (2) immunohistochemistry against Aβ 1–42. The results of the deconvolutions of the amide I band indicate that, ozone exposure causes a progressively decrease in the abundance percentage of α-helix secondary structure. Furthermore, the β-sheet secondary structure increases its abundance percentage. After 60 days of ozone exposure, the β-sheet band is identified in a similar wavenumber of the Aβ 1–42 peptide standard. Immunohistochemistry assays show an increase of Aβ 1–42 immunoreactivity, coinciding with the conformational changes observed in the Raman spectroscopy of Aβ 1–42 at 60 and 90 days. In conclusion, oxidative stress produces changes in the folding process of amyloid beta peptide structure in the dentate gyrus, leading to its conformational change in a final β-sheet structure. This is associated to an increase in Aβ 1–42 expression, similar to the one that happens in the brain of Alzheimer’s Disease (AD) patients.

## Introduction

Environmental pollution has become a public health issue in densely populated cities. One of the main pollutants resulting from photochemical air pollution is ozone. There is a large number of evidences that show the role played by ozone air pollution and its association to neurodegenerative diseases (Block and Calderón-Garcidueñas, 2009; Mumaw et al., 2016) such as Alzheimer’s Disease (AD; Moulton and Yang, 2012; Jung et al., 2015).

Exposition to ozone induces a state of oxidative stress if it is breathed chronically (Rivas-Arancibia et al., 2010). In previous works conducted in our laboratory, we have reported a non-invasive model of progressive neurodegeneration in rat hippocampus, caused by oxidative stress induced by low doses of ozone (Rivas-Arancibia et al., 2010; Rodríguez-Martínez et al., 2016).

Using this model in healthy rats, we have demonstrated that, without any other additional factors, oxidative stress by itself can produce damage and death cell along with the formation of Aβ 1–42, similar to what happens in AD patients (Rivas Arancibia et al., 2011; Hernández-Zimbrón and Rivas-Arancibia, 2014). In addition, ozone exposure also leads to a deficit in the learning and memory processes as well as motor and behavioral impairment in rats (Rivas-Arancibia et al., 1998; Dorado-Martínez et al., 2001).

The presence of Aβ 1–42 in the brain of AD patients is the final outcome of a series of metabolic changes that take place throughout extended periods of time during which the state of oxidative stress plays a key role. Replicating these changes in an animal model allows us to study the development of conformational alterations in peptides and proteins that occur during a chronic process of progressive neurodegeneration.

The peptide Aβ is generated by the cleavage of the amyloid precursor protein (APP) with the aid of α- and β-enzymes and γ-secretase (Hernández-Zimbrón and Rivas-Arancibia, 2014). The breakdown of APP occurs in two pathways: the non-amyloidogenic and amyloidogenic one. The amyloidogenic pathway is an alternative breakdown pathway of APP that leads to Aβ formation. The Aβ peptide of 40 amino acids (Aβ 1–40) is the most common (90%), while the 10% usually corresponds to the full-length Aβ 1–42. The latter is more hydrophobic and easily induces fibril formation.

Recent solid state NMR (ssNMR) studies have shed new light on Aβ 1–42 structure at atomic resolution (Xiao et al., 2015). They showed that triple parallel-β-sheet segments form the Aβ 1–42 with β-sheet motif, while Ahmed et al. (2010) proposed a mechanism to explain the conversion of Aβ 1–42 into fibrils. In addition, several molecular dynamics simulations have been performed in order to elucidate a possible mechanism in the conformational dynamics of Aβ 1–42. For example in different conditions, Aβ can aggregate in the form of monomers to oligos and finally in cytotoxic protofilaments of Aβ 1–42. In addition, there are studies that show the specific amino acid regions that favor the β-sheet assembly into insoluble protofilaments, in order to correlate these results with those obtained with NMR and X-ray diffraction experiments (Buchete et al., 2005; Zhu et al., 2012; Lee et al., 2016). However, neither the molecular mechanism nor the different structural conformations that Aβ 1–42 adopts, from the monomer phase to its final aggregation in insoluble protofibrils, have been established in animal models.

On the other hand, Raman spectroscopy has played an important role in the study of the influence of microenvironment on the biomolecular conformation, due to its ability to record the vibrational spectra of molecules in their biological environment even in animal models.

Even though, Raman spectroscopy cannot determine the molecular conformation of complex biomolecules as peptides or proteins at atomic level, it is highly informative for tracing the global conformational changes induced by modifications of its microenvironment. In general, the interpretation of the Raman results of biomolecules is based on the establishment of empirical correlations derived from structural studies performed with other techniques that provide information at atomic level, such as ssNMR, X-ray crystallography and molecular dynamics simulation (Zhang et al., 2000; Barth and Zscherp, 2002; Kong and Yu, 2007).

It is well known that Raman spectral intervals, known as amide regions, provide information on the changes that occur in the secondary structure of peptides and proteins (Uzunbajakava et al., 2003; Ruiz-Chica et al., 2004; Movasaghi et al., 2007; Bonnier and Byrne, 2012). In a normal Raman spectrum, there are three main regions that provide potential information on the vibrational modes of the proteins: amide I, II and III. Amide I band (1600–1700 cm^−1^), containing the in-plane vibrational mode of the C = O stretching and the N-H bending of the polypeptide bond, these modes are frequently used to follow the evolution of the peptide secondary conformational change. This band is mostly originated by vibrations from the amide groups found along the polypeptide skeleton, which are also sensitive to the Φ and ψ angles of each residue as well as the H-bonding pattern and peptide-peptide dipole coupling. The vibration of the amide modes is sensitive to the conformational changes of the polypeptide skeleton; it allows tracking the structural variations, which may be taking place in peptides and proteins during a progressive neurodegeneration process.

In addition, Raman spectroscopy has been reported in the literature as a tool to obtain information on biomolecules in the tissue of AD patients (Sudworth and Krasner, 2004; Chen et al., 2009; Tay et al., 2011; Park et al., 2013). These studies focus primarily on the exploration of the molecular conformation of insoluble Aβ 1–42 already formed, either chemically synthesized or obtained from patients with AD brains. However, to the best of our knowledge, there are no reports on the conformational changes that take place during the formation of Aβ 1–42 through the amyloidogenic pathway along the development of the progressive neurodegeneration process in an animal model.

In the literature, there are few works focusing on the conformational change of Aβ 1–42 under oxidative conditions. Some experiments have shown that methionine residues are very sensitive to oxidation, favoring a random coil structure in water at pH 4 (Watson et al., 1998; Friedemann et al., 2015). Some other reports have provided evidence that lipid oxidation favors the fibril aggregation of Aβ 1–42 into a β-sheet conformation (Boyd-Kimball et al., 2004; Butterfield et al., 2013; Korshavn et al., 2017). However, those results are not enough to establish a clear correlation to explain the steps followed during the conformational change of Aβ 1–42 in the AD, under oxidizing conditions.

The aim of this work is to study the effect of oxidative stress, caused by exposure to low doses of ozone, over the conformational changes of the secondary structure of Aβ 1–42, in the dentate gyrus of rat hippocampus in a model of progressive neurodegeneration.

## Experimental Procedure

### General Procedure

Animal care and handling were conducted in accordance with the National Institute of Health Guidelines for Animal Treatment, the Norma Oficial Mexicana NOM-036-SSA2-2002, and were approved by the Ethics Committee of the Medicine School at the National Autonomous University of Mexico.

Seventy-two male Wistar rats weighing 250–300 g were individually housed in acrylic boxes with free access to water and food (Purina, Minnetonka, MN, USA) and kept in a clear-air room. They were randomly divided into six experimental groups (*n* = 12 each): Group 1, exposed to an air stream free of ozone during 30 days; Group 2, exposed for 7 days to ozone; Group 3, exposed for 15 days to ozone; Group 4, exposed for 30 days to ozone; Group 5, exposed for 60 days to ozone; and Group 6, exposed for 90 days to ozone. The rats were exposed to ozone (0.25 ppm) daily for 4 h. Immediately after ozone exposure, the animals were returned to their home cages, and 2 h later they were processed for the following techniques: (1) Raman spectroscopy to follow the conformational structural changes of the Aβ peptides; and (2) immunohistochemistry Aβ 1–42.

### Ozone Exposure

Animals were put inside a chamber with a diffuser connected to a variable flux ozone generator (5 l/s) daily for 4 h. The procedure used has been described in other works (Pereyra-Muñoz et al., 2006; Rivas-Arancibia et al., 2010). The air feeding the ozone converter was filtered and purified. Ozone production levels were proportional to the current intensity and to the airflow. A PCI Ozone and Control System Monitor (West Caldwell, NJ, USA) was used to measure the ozone concentration inside the chamber throughout the experiment and to keep a constant ozone concentration.

### Air Exposure

The control group was treated in the same chamber described above using a flow of ozone-free purified air.

### Preparation of Tissue Samples for Raman Spectroscopy

After the last exposure to ozone, the rats were deeply anesthetized using pentobarbital sodium (50 ml/kg). The brains were extracted, perfused with 4% paraformaldehyde, dehydrated, and embedded in paraffin. Afterwards, 20 μm slices were cut using a microtome (SHUR/Cut2500, General Data Healthcare). The sections were deparaffinized and hydrated before conducting the spectroscopic analysis.

### Raman Spectroscopy Procedure

The deparaffinized sections were first examined by light microscopy to define a study region where six different cells were randomly selected. Five single spectra were recorded for each selected cell at different cytoplasm points close to the membrane cell. In this way, we obtained 30 single Raman spectra for each section.

Raman spectra were obtained using a Witec Alpha300 R confocal microscopy system, with a laser excitation source of 532 nm at a power of 0.7 mW, 600 l/mm grating, and a 50× plane Fluor objective with a numerical aperture of 0.45 at a working distance of 4.5 mm. The spectral resolution used was 4 cm^−1^ while a 576 × 400 pixel thermoelectric-cooled charge-coupled device (CCD) was used as detector.

Before each measurement, a system calibration was performed using a silicon chip in order to check the standard band position and the intensity. Data were measured over the 200–4000 cm^−1^ spectral range. Each spectrum consisted of five accumulations with an integration time of 0.5 s to avoid tissue damage. As a reference, we used pure Aβ 1–42 (Sigma-Aldrich) to obtain its Raman spectra under similar experimental conditions.

### Spectral Analysis

The spectra were pre-processed for base line correction and cosmic ray removal using WITEC Project 2.0 software. After that, the 30 spectra obtained from each tissue section under study were vector-normalized with Wolfram-Mathematica software and, finally, each set of 30 spectra was averaged. This procedure was used for the control group as well as the groups exposed to ozone for 7, 15, 30, 60 and 90 days.

Additionally, a deconvolution of the amide I band (1600–1700 cm^−1^) of the spectra was performed in order to visualize the subjacent sub-bands that compose the whole complex amide I band. The original spectra were baseline-corrected before the deconvolution analysis and smoothed with the Savitzky-Golay (SG) filter with five-point window. The deconvolution was performed using Origin 6.0 software, selecting the Gaussian peak type option. The number of sub-bands employed for this spectral analysis was defined by the second derivative of the whole amide I band. In addition, two bands at ~1604 cm^−1^ and ~1612 cm^−1^ were included with the amide I band during the deconvolution process since they were not baseline separated from the amide I feature. The values of the area under the curve of each sub-band were taken, in a first approximation, as proportional to the relative abundances of the peptide motifs associated with each sub-band.

### Immunohistochemistry Technique

Six animals from each group were anesthetized with pentobarbital sodium (50 mg/kg i.p.; Sedalpharma, Edo. de México, Mexico) and transcardially perfused with 4% paraformaldehyde (Sigma-Aldrich Chemie, Germany) in 0.1 M phosphate buffer (J.T Bakker E.U., New Jersey, Kentucky and Mexico; PB, Tecsiquim; pH 7.4). The brains were post fixed with 10% formaldehyde (J.T Bakker U.S.A., New Jersey, Kentucky and Mexico) for 24 h and embedded in paraffin (McCormick, St. Louis, MO, USA). Sagittal slices of the brain containing the hippocampi were cut at 5 μm on a microtome (ShurCut 2500. General Data Healthcare) and mounted on slides. Slices of each brain containing the hippocampi were deparaffinized and pretreated with heat retrieval solution (Biocare Medical, Concord CA, USA) and introduced into an electric pressure cooker (Decloacking Chamber, Biocare Medical) for 20 min. After being washed with distilled water and treated with 3% hydrogen peroxide (J.T Baker U.S.A., New Jersey, Kentucky and Mexico) for 5 min, the slices were rinsed again and treated with a blocking reagent (Background Sniper, Biocare Medical, Concord, CA, USA) for 10 min. They were washed with saline phosphate buffer (PBS, Merck, Germany) and incubated overnight at 4°C with Aβ 1–42 (purified monoclonal mouse antibody, diluted 1:50 (ANASPEC, Fremont, CA, USA). Sections were rinsed with PBS and treated with secondary antibody using Trekkie Universal Link (Starr Trek Universal HRP Detection; Biocare Medical) for 1 h. The sections were later washed with PBS, and then treated with Trekavidin–HRP Label (Starr Trek Universal HRP Detection, Biocare Medical) for 30 min. Bound antibody was visualized using 3,3′–Diaminobenzidine (DAB Substrate Kit, ScyTek, Logan, UT, USA) as the chromogen. The slices were washed with distilled water and contrasted with hematoxylin buffer solution (Concord, CA, USA). Representative brain slices from each group were processed in parallel. Following cover slipping with Entellan (F/550 ml Merck, Germany), the slices were examined with an Olympus BX41 Microscope (Japan) and photographed (Evolution VF-F-CLR-12 Media Cybernetics camera; Bethesda, MD, USA).

## Results

### Raman Spectra of Rat Hippocampus Tissue

**Figure 1:**
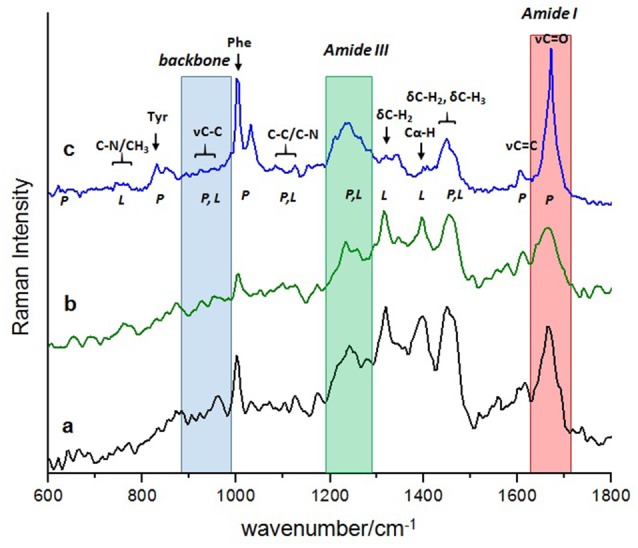
**Comparison of the complex Raman “fingerprint” spectral region between: the rat hippocampus tissue at the beginning of exposure, control (a); at the end of exposure, 90 days (b), with the less complex Raman spectra of the Aβ 1–42 pure peptide, reference (c)**. Normal vibrational modes of biomolecules are assigned and indicated in the wavenumber region where usually appear. Shadow regions indicate the commonly vibrational modes of proteins used to follow the evolution of its secondary structure at a conformational level: red; the amide I band; green, the amide III band and blue, the skeletal backbone vibration C-C. Abbreviations: υ (stretching mode), δ (deformation mode), P (proteins) and L (lipids), Tyr (tyrosine), Phe (phenylalanine).

The Raman spectra of the “finger-print” region (600–1800 cm^−1^) of rat hippocampus tissue were analyzed (Figure 1). The spectra presented correspond to the control groups (Figure 1a) and the group exposed to ozone for 90 days (Figure 1b), together with that of the pure Aβ 1–42 peptide used as a reference (Figure 1c). The complexity of the Raman spectra, both from a tissue (a and b) and from a pure peptide sample (c), is seen in a comparative way in Figure 1.

The regions conventionally used to make a structural study of proteins based on their Raman spectrum are amide I, amide III and skeletal backbone vibration C-C, which are shaded in Figure 1. It is important to note that the amide band I (1640–1680 cm^−1^) of each spectrum presents a different profile in relation to bandwidth, wavenumber and intensity. From a direct observation of this band, it is not possible to estimate the dominant secondary conformation in each case. To achieve this purpose, the profile of the amide I band must be mathematically processed by a deconvolution analysis to obtain this information.

The regions of amide III (1200–1340 cm^−1^) and skeletal backbone vibration C-C (890–980 cm^−1^) help, in principle, to corroborate the secondary structure derived from the analysis of the amide I band. However, many other vibrational modes are also in this region, which come from other biomolecules that are present in the rat tissue and overlap with each other. This makes it difficult, in our case, to use these regions (amide III and skeletal backbone vibration C-C) to confirm the secondary structure of the Aβ 1–42 in our results.

### Raman Spectrum of Reference Aβ 1–42 Peptide

**Figure 2:**
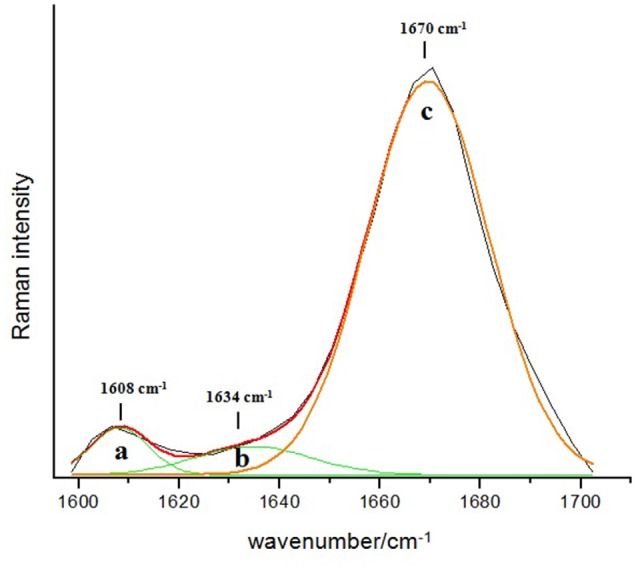
**Deconvolution of the amide I band region (1600–1700 cm^−1^) in the Raman spectrum of the reference Aβ 1–42**. The dominant band at 1670 cm^−1^ with 89% of relative abundance (c) indicates the presence of the β-sheet secondary structure type, while the two minor bands at 1608 cm^−1^ (a) and 1634 cm^−1^ (b) are assigned to the amino acids side chains (6%) and the aromatic amino acids ring mode (5%), respectively.

The Raman spectrum of the commercial Aβ 1–42 peptide that is used as a reference for the experimental direct identification of the β-sheet conformation in the amide I band of this peptide is shown in Figure 2. The spectrum is dominated by a Gaussian type band centered at 1670 cm^−1^ (Figure 2c) which is assigned mainly to the C = O stretching vibration of the β-sheet secondary structure in the Aβ 1–42 peptide (Barth and Zscherp, 2002; Rygula et al., 2013). Additionally, the deconvolution of the spectral region between 1600 cm^−1^ and 1700 cm^−1^ shows two minor bands: 1608 cm^−1^ (Figure 2a) and 1634 cm^−1^ (Figure 2b), which are assigned to the amino acid side chains and the aromatic amino acid ring mode vibration, respectively (Rahmelow et al., 1998; Rygula et al., 2013). The percentage of area under the curve for each of these bands is 89% for the β-sheet structure, 6% for the amino acid side chains and 5% for the aromatic amino acid ring mode vibration.

### Raman Spectra of Tissue from the Different Rat Groups, Control and Those Exposed to Different Periods of Ozone

**Figure 3:**
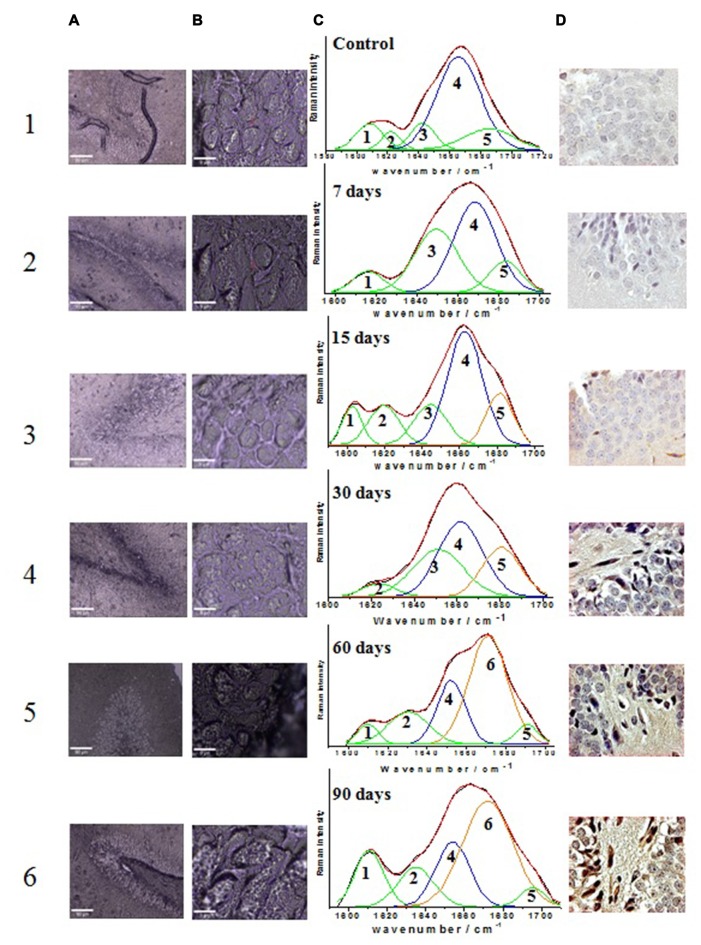
**Effect of exposure to low doses of ozone on the relative abundance of different secondary conformations of the Aβ 1–42 peptide**. Columns **(A)** and **(B)** show the micrographs of the areas where the punctual Raman spectra were obtained (**A**: 10×; **B**: 100×); column **(C)** shows the tissue Raman spectra in the amide I spectral interval (1600–1720 cm^−1^); column **(D)** shows the micrographs of immunoreactivity of Aβ 1–42 peptide in the dentate gyrus of hippocampus (40×). Row 1 shows the results of the control group while rows 2, 3, 4, 5 and 6 show the groups exposed to ozone at 7, 15, 30, 60 and 90 days, respectively. The assignments of the spectral sub-bands are presented in Table 1. The arrows on immunoreactivity micrographs indicate the Aβ 1–42 expression.

Confocal micrographs of hippocampal cells where the Raman spectra were obtained (Figure 3, columns **A** and **B**) along with the results from the mathematical deconvolutions of the amide I band (Figure 3, column **C**). The micrographs of the immunohistochemistry against Aβ 1–42 for each of the experimental groups (control and those exposed to ozone for 7, 15, 30, 60 and 90 days) are also shown (Figure 3, column **D**).

The deconvolution of the amide I band (Figures 3C1–C6) for the groups evidences the presence of vibrational modes assigned as follows: 1602–1610 cm^−1^ amino acid side chains vibrations (Rahmelow et al., 1998), 1615–1635 cm^−1^ aromatic amino acid ring mode vibrations (Rygula et al., 2013), 1644–1650 cm^−1^ random coil structure (Sekhar et al., 2015), 1655–1668 cm^−1^ α-helix secondary structure (Lippert et al., 1976; Rygula et al., 2013; Kurouski et al., 2015), 1670–1682 cm^−1^ β-sheet secondary structure (Mahadevan-Jansen and Richards-Kortum, 1996; Wen, 2007; Kurouski et al., 2015), and 1683–1685 cm^−1^ β-turn structural motifs (Barth and Zscherp, 2002). Table 1 summarizes the distribution of the sub-bands resulting from the deconvolution analysis for the control and all the ozone-exposed groups, indicating the percentage relative abundance and the assignment for each Raman vibrational mode.

**Table 1:** **Deconvolution of the amide I mode of different proteins and peptides along exposure time**.

Band number	Center (cm^−1^)	Band area	Abundance (%)	Assignment
**Control group, no exposure to ozone**				
1	1608	0.073	10	Amino acids side chains
2	1622	0.036	5	Aromatic amino acids ring mode
3	1642	0.069	10	Unordered structure
4	1665	0.043	60	α-helix
5	1685	0.108	15	β-turn
**Group exposed to ozone for 7 days**				
1	1615	0.068	8	Amino acids side chains
3	1649	0.292	33	Unordered structure
4	1668	0.405	47	α-helix
5	1683	0.106	12	β-turn
**Group exposed to ozone for 15 days**				
1	1602	0.078	9	Amino acids side chains
2	1619	0.124	13	Aromatic amino acids ring mode
3	1644	0.140	16	Unordered structure
4	1662	0.412	46	α-helix
5	1681	0.143	16	β-turn
**Group exposed to ozone for 30 days**				
2	1623	0.051	6	Aromatic amino acids ring mode
3	1650	0.277	30	Unordered structure
4	1661	0.382	41	α-helix
5	1680	0.218	23	β-turn
**Group exposed to ozone for 60 days**				
1	1609	0.037	5	Amino acids side chains
2	1630	0.118	16	Aromatic amino acids ring mode
4	1652	0.160	22	α-helix
6	1671	0.376	52	β-sheet
5	1690	0.038	5	β-turn
**Group exposed to ozone for 90 days**				
1	1610	0.143	15	Amino acids side chains
2	1635	0.125	13	Aromatic amino acids ring mode
4	1655	0.196	20	α-helix
6	1672	0.047	48	β-sheet
5	1695	0.042	4	β-turn

In the control group the amide I band (Figure 3C1), is mostly composed by the corresponding α-helix peptide secondary structure (60%) (band 4), this band is centered at 1665 cm^−1^. Additionally, there is a minor contribution of the vibrational motifs of the amino acids side chain (10%) (band 1), the aromatic amino acids ring mode (band 2) (5%), the unordered structure (10%) (band 3) and the β-turn type structure (15%) (band 5). On the other hand, the micrograph of the dentate gyrus shows absence of reactivity to Aβ 1–42 by immunohistochemistry (Figure 3D1).

The deconvolution of the amide I band for the groups exposed to ozone for 7 days (Figure 3C2), this band still shows a clear majority of the α-helix secondary structure conformation (47%) now centered at 1668 cm^−1^ (band 4). Additionally, there is an increase in the abundance of the bands corresponding to the unordered structure (33%) (band 3), the vibrational modes of amino acids side chain (8%) (band 1), and the β-turn structure (12%) (band 5). For its part, the immunohistochemistry assay shows absence of immunoreactivity against Aβ 1–42 (Figure 3D2).

At 15 days of ozone exposure (Figure 3C3), there is an evident change in the amide I band profile, particularly in the region of the high wavenumbers where the formation of a shoulder at 1680 cm^−1^ is observed (band 5); these changes are reflected in the deconvolution sub-bands. The predominant band is still the one corresponding to the α-helix secondary structure (45%), centered now at 1662 cm^−1^ (band 4), together with the vibrations of the amino acids side chain (9%) (band 1) and the aromatic amino acid ring mode (13%) (band 2). The most significant change for this period of exposure is observed in the band associated to the β-turn structure (band 5), which increases its relative abundance (16%) and moves towards slightly lower wave numbers centering at 1681 cm^−1^. For its part, in the microphotograph of the dentate gyrus in the hippocampus, the beginning of a diffuse extracellular immunoreactivity for Aβ 1–42 is observed (Figure 3D3).

In the group exposed to ozone for 30 days (Figure 3C4), the amide I band profile still shows the change in its profile for the high wavenumber side. The corresponding deconvolution now shows four sub-bands: one associated to the aromatic amino acids ring mode (6%) (band 2), the unordered secondary structure (30%) (band 3) and the α-helix secondary structure (41%) (band 4). The sub-band corresponding to the β-turn structures increases its abundance (23%) (band 5) and continues moving toward low wavenumbers. On the other hand, in the corresponding micrograph of the dentate gyrus in the hippocampus, Aβ 1–42 shows a clear increase of intracellular immunoreactivity, together with cell edema and extracellular diffuse immunoreactivity (Figure 3D4).

After 60 days of ozone exposure (Figure 3C5), the changes in the amide I band envelope are even more evident, a fact which is reflected in the relevant changes in the result of its deconvolution. Indeed, the sub-band corresponding to the α-helix secondary structure (band 4) considerably decreases its abundance (22%) while band 6 becomes predominant (52%) and is now centered at 1671 cm^−1^, in clear accordance with the wavenumber of the previously assigned β-sheet structure of pure Aβ 1–42 presented in Figure 2. In a similarly evident way, the β-turn band (band 5) shows a significant decrease in its abundance (5%) and a deviation toward higher wavenumbers (1690 cm^−1^). For their part, the bands of the amino acids side chains (band 1) and the mode of the aromatic amino acids ring (band 2) remain unchanged. In agreement with these spectral changes, the immunohistochemistry in the dentate gyrus of the hippocampus shows an intracellular increase of Aβ 1–42 immunoreactivity, which is also evident in the neuropil, together with cell swelling and pyknotic nuclei (Figure 3D5).

The deconvolution of the amide I band for the group exposed to ozone for 90 days (Figure 3C6) shows a similar distribution to that of the previous group. Once again, the most abundant sub-band (48%) corresponds to the β-sheet secondary structure (band 6) centered at 1672 cm^−1^. The representative sub-band of the α-helix secondary structure (band 4) at 1655 cm^−1^, decreases its relative abundance to 20%, while the one of the β-turn structure (band 5), centered at 1695 cm^−1^, remains around 4%. Moreover, the sub-bands corresponding to the side chains of aromatic amino acids (band 1) and the breathing mode of the aromatic amino acids ring (band 2) have now a contribution of 15% and 13%, respectively. Once again, consistent with the spectral results, the micrograph of the dentate gyrus of the hippocampus shows increased Aβ 1–42 immunoreactivity, both intracellularly and at the neuropil, with morphological changes as swelling and pyknotic nuclei (Figure 3D6).

In relation with the optical micrographs of columns A and B in Figure 3, which shows the areas where the punctual Raman spectra were obtained, it is important to note the alteration of the morphology of neurons, in particular of the cell membrane, in correlation with the increase in the time of exposure to ozone. These observations are consistent with the results of the immunohistochemistry against Aβ 1–42 presented in column D of Figure 2.

### Evolution of the Percentage of Abundance of the Secondary Structure Motifs of the Amide I Band

**Figure 4:**
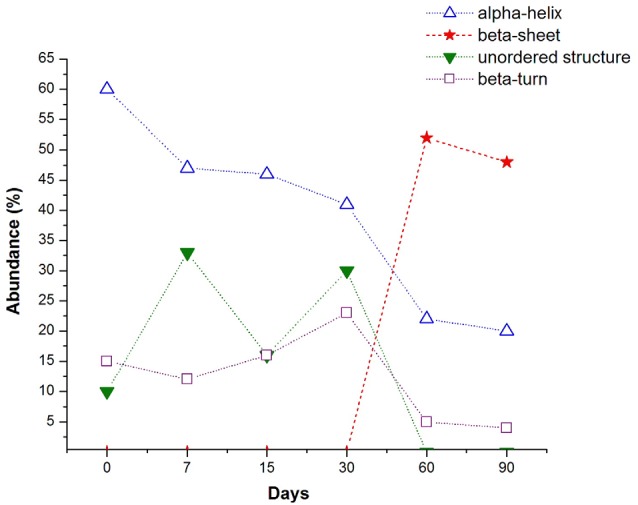
**Evolution of the percentage of abundance of the main secondary structure motifs found in the deconvolution of the amide I band, along the days of ozone exposure (dashed lines are guide for the eyes)**. The α-helix conformation experiments a continuous decline when the ozone exposure time increases, the most pronounce decrement corresponds with the abrupt increase of the β-sheet conformation. The β-turn type slightly increases until 30 days and then decreases with a similar tendency to the α-helix motif. Unordered structure type shows a random tendency but almost disappears after 60 days of ozone exposure, when the β-sheet conformation is the most abundant.

Finally, in Figure 4 we show a general view of the trend of the major conformational secondary Aβ 1–42 motifs, resulting from the deconvolution analysis of the amide I band of the hippocampal tissue of rats, exposed to low doses of ozone for different periods. It is clear how the α-helix motif experiments a continuous decrease as a function of the increase ozone exposure time. The more pronounced decrease of the α-helix band between 30 and 60 days of ozone exposure, corresponds with the appearance and the strong increase of the band at 1671 cm^−1^, associated with the β-sheet motif of the Aβ 1–42 sary structure. After 60 days of ozone exposure, the relative abundance of the α-helix and β-turn motifs seems to reach an almost steady state. On the other hand, there is not a clear trend in the changes of the β-turn and the non-ordered structures during the first 30 days of exposure to ozone. However, the near disappearance of these bands after 60 days of exposure seems to be correlated with the strong growth of the β-sheet secondary structure during the same period.

### Number of Cells Expressing Aβ 1–42 in Hippocampus of Rats Chronically Exposed to Ozone

**Figure 5:**
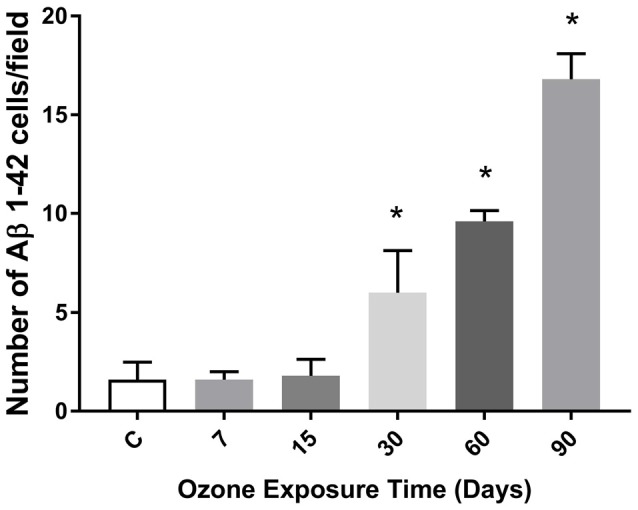
**Effects of oxidative stress on the number of immunoreactive cells to Aβ 1–42 depending of the time of exposure to ozone in the hippocampus**. The graph shows an increase of the cell number positive to Aβ 1–42 at 30, 60 and 90 days of ozone treatment (*p < 0.05).

The chronic exposure to low doses of ozone leads to an increase in the number of cells expressing the Aβ 1–42 peptide from 30 to 90 days of exposure (Figure 5). Also, we can also observe that the average number of cells immunoreactive to beta-amyloid 1–42, increases in accordance with the increase of the abundance of the beta-sheet structure from 30 to 60 days, presented previously in Figure 4.

## Discussion

The Raman spectroscopy results presented in this work, show a temporal evolution of the different structural motifs, mainly α-helix and β-sheet, that conform the secondary structure of the Aβ 1–42 in hippocampus of rats exposed to low doses of ozone for different periods of time.

To the best of our knowledge, these results report, for the first time, the effect of chronic oxidative stress on the conformational secondary structure of Aβ 1–42 of hippocampal tissues of rats exposed to low doses of ozone. A predominance of α-helix structures in the hippocampus of control animals is clearly established as the initial condition. As the neurodegeneration process advances, from the initial step until 30 days, the α-helix structure seems to be destabilized in favor of β-turn and unordered structures. Subsequently, between 30 and 60 days, when the process of neurodegeneration has set in, the β-sheet secondary structure is observed to appear and grow at the expense of the β-turn and unordered structural motifs. Finally, after 60 days of ozone exposure, the β-sheet motif remains the predominant secondary structure in the Aβ 1–42 peptide. The formed scenario is reinforced by immunohistochemistry studies of hippocampal tissue slices of animals that were treated with low doses of ozone for 60 and 90 days, showing high immunoreactivity against Aβ 1–42.

As described above, we tracked the evolution of the sub-bands that create the amide I spectral interval and its association to the immunohistochemistry response toward Aβ 1–42. This allowed us to distinguish different stages in the temporal evolution of the existing sub-bands in the tissue of the dentate gyrus in the hippocampus of rats exposed to low doses of ozone (Figure 3).

The first stage covers the groups exposed to ozone for 7 and 15 days. The Raman spectra of this stage show a decrease of the sub-band corresponding to the conformation of α-helix along with a considerable variability of the sub-bands of unordered and β-turn structural motifs (Figure 4). All this suggests the beginning of the destabilization of the α-helix structure that, in consequence, gives way to other secondary structural motifs.

This first stage may be considered to represent an initial process of the conformational changes, even though other studies demonstrate that, during this stage, there is an increase of lipid oxidation and oxidized proteins, as well as an increase in inflammation markers (Rivas-Arancibia et al., 2010; Rodríguez-Martínez et al., 2013).

The second stage corresponds to the group exposed to ozone for 30 days, and it is defined by the onset of Aβ 1–42 intracellular immunoreactivity. It is spectrally accompanied by a significant growth of the sub-bands associated to unordered structures and β-turn, as well as an additional decrease of the relative abundance of the α-helix secondary structure. Along with the previous changes, there is also a greater increase of oxidized lipids and proteins. In our model, these results indicate that the alterations are irreversible.

The third stage corresponds to the groups exposed for 60 and 90 days. In which, we observe an additional increase of immunoreactivity, both intracellularly and at the neuropil, together with cell swelling and pyknotic nuclei. This is spectrally associated with the emergence of a new predominant sub-band corresponding to the β-sheet secondary structure motif together with an additional reduction in the abundance of the sub-bands of α-helix, and unordered and β-turn motifs. In this stage, the reduction of the β-turn and α-helix structures seems to precede the formation of the β-sheet motifs, the predominant secondary structure of Aβ 1–42.

**Figure 6:**
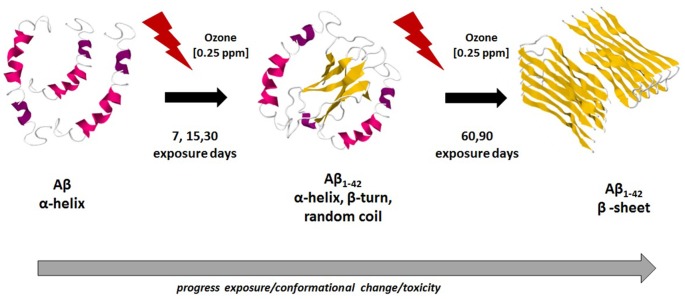
**Hypothetical schematic representation of conformational changes of Aβ 1–42 along ozone exposure**. In the first stage, there is an abundance of the α-helix conformation. After 30 days of ozone exposure, in a second stage, there is a mixture of α-helix, β-turn and random coil motifs. After 90 days of ozone exposure, the third stage, there is a majority of β-sheet secondary structure conformation. This trend is related with the increase toxicity of ozone and the neurodegeneration progress in the hippocampus of rats. Model figures 1Z0Q, 2OTK and 2BEG, taken from the PDB files.

These results are in agreement with general conclusions already established by other works, which used different methodologies and techniques (Soto et al., 1995; Kirkitadze et al., 2001), indicating that the Aβ 1–42 original α-helical conformation transforms into β-sheet, which finally results in the fibrillar Aβ 1–42. Our results also indicate that the Aβ peptide first forms a transient α-helical conformation and then, when the neurodegeneration process progresses, there is an increase in the β-sheet conformation which is characteristic of Aβ 1–42 fibrillar. In this framework, the major contribution of this work is to provide a deeper understanding of the evolution of the structural motifs that shape the secondary structure of Aβ 1–42 from the original α-helical conformation to the β-sheet structures. The results found in this work are summarized in Figure 6, which presents the hypothetical schematic representation of the conformational changes of Aβ 1–42 peptide in hippocampal tissue of rats exposed to low ozone doses, this figure was bases on the published structures obtained from different techniques (Lührs et al., 2005; Tomaselli et al., 2006; Hoyer et al., 2008).

On the other hand, there exists a clear process of progressive neurodegeneration followed by a major increase of oxidized biomolecules, mitochondrial deficit and a decrease of antioxidant systems (Rodríguez-Martínez et al., 2013). The above may indicate that, unlike Aβ 1–40, which is commonly formed in the brain (Yin et al., 2007), Aβ 1–42 shows a change in its spatial conformation, promoted by oxidative stress (Butterfield et al., 2013; Korshavn et al., 2017). Aβ 1–42 is an oxidized peptide that changes its spatial conformation from β-turn to β-sheet as proven by this work. This peptide may act as a reactive species since it leads to the oxidation of neighboring molecules as many other researchers have demonstrated (Butterfield et al., 2013). This indicates that the role of oxidative stress is fundamental in Aβ 1–42 formation and during the maintenance of the process of progressive neurodegeneration. The intracellular increase of Aβ 1–42 as well as its entry to the mitochondrion (Hernández-Zimbrón and Rivas-Arancibia, 2015) helps keeping the damage since it inhibits ATP production, which is necessary to repair and maintain cell metabolism (Rodríguez-Martínez et al., 2013). It also hampers the correct function of the endoplasmic reticulum as neurodegeneration progresses and leads to an increase of protein misfolding, which contributes to accumulate intracellular oxidized proteins leading to cell death (Rodríguez-Martínez et al., 2016).

Something similar may be occurring in AD since its diagnosis is done when the disease is advanced. Similarly, Aβ 1–42 plaques seem to be the final outcome of a long process of pathophysiological changes and increase in the production of reactive species (Butterfield et al., 2013). These species work as signals to attract phagocytic microglia cells (Rivas-Arancibia et al., 2010), macrophages and others, increasing the oxidative stress. Although several studies have shown that there are alterations in Aβ 1–42 fibers, the mechanisms through which Aβ 1–42 is progressively formed in the brain are still unclear. This study allows us to demonstrate how the conformation of the peptide progressively changes from α-helix to β-turn plus unordered structure, and finally to β-sheet secondary structure. This might explain why inhibiting the formation of Aβ 1–42 plaques cannot restore the normal functioning of the brain tissue and that it is necessary to counter the factors that contribute to neuronal damage, like oxidative stress.

## Conclusions

The formation of Aβ 1–42 is promoted in a murine model of progressive neurodegeneration similar to AD, caused by repeated exposure to low doses of ozone, which induces a state of chronic oxidative stress. This peptide shows conformational changes from α-helix to a mixture of α-helix, β-turn and unordered structure, leading to a β-sheet secondary structure. This promotes polymerization, misfolding and protofibrill aggregation of Aβ 1–42 in neurotoxic and insoluble plaques. These conformational changes in the secondary peptide structures may be the key to understand the formation of both intra and extracellular Aβ 1–42 deposits, similar to what could be happening in the brain of AD patients.

## Author Contributions

SR-A proposed the objective of this work, designed the experimental strategy, contributed with the analysis, interpretation and discussion of the results obtained for this study and work on the writing of the manuscript. ER-M, carried out the techniques of immunohistochemistry, did Figure 2, contributed with the analysis, interpretation and discussion of the results obtained for this study and work on the writing of the manuscript. IB-R, carried out the Raman records, the analysis of the same and the realization of Figure 3 and Table 1, contributed with the analysis, interpretation and discussion of the results obtained for this study and work in the writing of the manuscript. UL-G, participated in the realization of Raman records, the analysis of the same and the realization of Figure 3. He contributed with the analysis, interpretation and discussion of the results obtained for this study and work on the writing of the manuscript. JMS participated in the design of the experimental strategy, contributed with the analysis, interpretation and discussion of the Raman results obtained for this study and work on the writing of the manuscript.

## Conflict of Interest Statement

The authors declare that the research was conducted in the absence of any commercial or financial relationships that could be construed as a potential conflict of interest.
